# Specific modes of exercise to improve rotator cuff-related shoulder pain: systematic review and meta-analysis

**DOI:** 10.3389/fbioe.2025.1560597

**Published:** 2025-04-08

**Authors:** Dianxuan Wu, Zhicheng Wen, Haolin Ke, Jiexin Zhang, Shaozi Zhong, Jiachen Teng, Lan Xu, Jintao Li, Yan Shao, Chun Zeng

**Affiliations:** ^1^ Department of Sports Medicine, Center for Orthopaedic Surgery, The Third Affiliated Hospital of Southern Medical University, Guangzhou, China; ^2^ Department of Orthopedics, Orthopedic Hospital of Guangdong Province, Academy of Orthopedics·Guangdong Province, The Third Affiliated Hospital of Southern Medical University, Guangzhou, China; ^3^ The Third School of Clinical Medicine, Southern Medical University, Guangzhou, China; ^4^ Guangdong Provincial Key Laboratory of Bone and Joint Degeneration Diseases, Department of Cell Biology, School of Basic Medical Sciences, Southern Medical University, Guangzhou, China; ^5^ The Second School of Clinical Medicine, Zhujiang Hospital, Southern Medical University, Guangzhou, China

**Keywords:** exercise therapy, specific exercise, chronic sports injuries, rotator cuff-related shoulder pain, meta-analysis

## Abstract

**Objective:**

To investigate the effect of specific modes of exercise on rotator cuff-related shoulder pain (RCRSP) patients, aiming to provide a theoretical reference for conservative treatment and exercise prescription.

**Methods:**

Data sources included PubMed/MEDLINE, Web of Science, ScienceDirect, and CNKI, covering studies from database inception to June 2024. Study selection followed pre-set inclusion/exclusion criteria. Cochrane methods guided quality assessment and data extraction. Outcome measures included VAS, CMS, and DASH scores. Publication bias assessed via funnel plots; forest plots created using RevMan 5.4.

**Results:**

13 studies (n = 690) met inclusion criteria for RCRSP exercise interventions. It is indicated that: (1) specific exercises led to modest improvements in performance of pain (SMD = −0.31, 95% CI: 0.46 to −0.16, P < 0.0001) and function, with enhancements in CMS (SMD = 0.59, 95% CI: 0.44 to 0.74, P < 0.00001) and DASH (SMD = −0.60, 95% CI: 0.80 to −0.39, P < 0.00001). (2) Interventions lasting less than 2 months were slightly more effective than those lasting 2 months or longer, observed in VAS (SMD = −0.35, 95% CI: 0.56 to −0.15, P = 0.0007), CMS (SMD = 0.71, 95% CI: 0.47 to 0.96, P < 0.00001), and DASH (SMD = −0.71, 95% CI: 0.99 to −0.43, P < 0.00001). (3) Subgroup analyses revealed that handgrip strength exercises enhanced shoulder pain relief, shoulder mobilization/stretching improved both function and pain levels over 2 months, scapular stabilization exercise improved pain and DASH performance over 2 months, eccentric exercises boosted CMS and DASH performance over 2 months, while proprioceptive exercises showed no significant improvement in pain or CMS performance.

**Conclusion:**

Compared to non-specific exercises, specific exercise programs moderately alleviate RCRSP symptoms, with shorter interventions (<2 months) demonstrating marginally superior outcomes. Efficacy varies by exercise type, emphasizing the need for individualized prescriptions.

**Systematic Review Registration:**

PROSPERO (CRD42024550602).

## 1 Introduction

The shoulder joint, characterized by its complex interplay of bones, muscles, ligaments, and nerves, is highly mobile. Pain in this area is a prevalent musculoskeletal disorder, affecting up to 70% of individuals over their lifetime (Requejo-Salinas et al., 2022). Prognosis varies widely, with up to 50% of patients experiencing persistent pain 6–12 months after seeking clinical treatment, significantly impacting their work and daily life (Pope et al., 1997; Picavet and Schouten, 2003; Greving et al., 2012; Doiron-Cadrin et al., 2020). The complex patho-anatomy of the area complicates traditional diagnosis and pinpointing a specific pain source (Greenberg, 2014; Gismervik et al., 2017). Rotator cuff-related shoulder pain (RCRSP) is an umbrella term that encompasses a range of shoulder conditions including subacromial pain (impingement) syndrome, rotator cuff tendinopathy, symptomatic partial and full thickness rotator cuff tears, and is the most common cause of shoulder pain (Lewis, 2016; Requejo-Salinas et al., 2022). Characteristic clinical manifestations include painful shoulder elevation/external rotation with concomitant motion restriction and functional impairment. Therapeutic objectives prioritize restoration of pain-free range of motion and optimal shoulder function (Ottenheijm et al., 2011).

The sources of pain in RCRSP are primarily related to decreased joint function, decreased muscle performance, altered shoulder kinematics, and maladaptive pain behaviors such as agoraphobia and exaggeration, and treatment consists of: patient education, exercise therapy, medications, joint cavity injections, and surgical interventions (Dubé et al., 2023). Exercise therapy is a cornerstone in treating shoulder pathology, with numerous reviews highlighting its effectiveness in improving pain and function (Kromer et al., 2009; Abdulla et al., 2015; Steuri et al., 2017; Powell et al., 2024), which takes the form of a physical therapist-initiated, patient-guided strengthening exercise through antigravity exercise followed by progressive resistance exercise, with the patient following a self-management program at home (Greenberg, 2014; McInnis and Morehead, 2020).

The efficacy of exercise therapy is well-established, yet a consensus on the optimal modalities and exercise types remains elusive. Shoulder-specific exercise therapy is distinguished by its targeted, biomechanically-driven approach that simultaneously activates scapulothoracic musculature and glenohumeral stabilizers. This approach encompasses various components, including scapular stabilization, positioning, proprioception, neuromuscular control, strength and extension, all integral to enhancing shoulder joint function (Hanratty et al., 2012; Holmgren et al., 2014; Hanratty et al., 2016). While articles have investigated the effectiveness of exercise-specific therapy for adolescent scoliosis (Xin-jing et al., 2024), there remains a relative paucity of research exploring the efficacy of shoulder-specific exercise therapy.

## 2 Methods

This systematic review was previously registered with the International Prospective Register of Systematic Reviews (PROSPERO CRD: 42024550602) and adheres to the guidelines set forth by the Cochrane Collaboration (Julian et al., 2019).

### 2.1 Search strategy

Searches across five databases: PubMed/MEDLINE, Web of Science, ScienceDirect, and CNKI were conducted for studies on exercise therapy for rotator cuff-related shoulder pain published from the inception of each database through June 2024. The search terms for databases included exercise, exercise therapy, exercise prescription, training, kinematics, shoulder pain, rotator cuff-related shoulder pain, subacromial impingement syndrome, rotator cuff injury, and rotator cuff tendinopathy. The controlled descriptors and keywords were combined with the ‘AND’ and ‘OR’ Boolean operators when appropriate.

### 2.2 Inclusion and exclusion criteria

For inclusion, studies were required to involve patients with clinically diagnosed rotator cuff-related shoulder pain (RCRSP), including conditions such as subacromial impingement/pain, rotator cuff injury, and rotator cuff tendinopathy. The intervention group was required to undergo specific exercise therapy, which could include individualized, supervised, progressive, or targeted exercises, or exercise combined with conventional treatments. The control group was to receive other conventional exercise interventions distinct from those in the intervention group. Outcome measures had to include at least one of the following: Pain Visual Analogue Scale (VAS) to assess shoulder pain; Constant-Murley Score (CMS) or Disability of the Arm, Shoulder and Hand Score (DASH) to assess shoulder functional status. The literature types considered were randomized controlled trials published nationally and internationally.

Exclusion criteria included studies involving patients with shoulder pain not related to rotator cuff issues (e.g., frozen shoulder) or those who had undergone shoulder surgery. Additionally, studies were excluded if the full text was unavailable, if data were incomplete, or if the literature was duplicated.

### 2.3 Data extraction

Data extraction was carried out by one researcher and subsequently verified for accuracy by a second researcher. Any disagreements were resolved by a third researcher, who facilitated discussions with the first two researchers to reach a consensus. Multi-arm studies were partitioned into multiple two-arm comparisons in accordance with a pre-established table detailing the characteristics of the included studies. This table included details such as: first author’s name, year of publication, characteristics of the study population (diagnosis, sample size, age, gender), intervention details (experimental group, control group), time to assessment (with baseline data collected after the start of the intervention), outcome.

### 2.4 Risk of bias assessment

Risk of bias assessment was performed according to the principles of the Cochrane Handbook and operated through Review Manager 5.4 software. Seven indicators of quality were evaluated for the included literature, including: randomized allocation scheme, concealed allocation scheme, blinding method, blinding for outcome assessment, completeness of outcome data, selective reporting of study results, and other biases. The results of bias evaluation for each item were categorized as low risk (green), high risk (red), and unclear risk (yellow).

### 2.5 Data analysis

The article was subjected to traditional meta-analysis and quality assessment using RevMan 5.4 software. Statistical heterogeneity was evaluated with the I^2^ test, considering the interventions and outcome measures employed. For studies with low statistical heterogeneity (I^2^≦50%), the fixed-effects model was applied; otherwise, the random-effects model was used. Outcomes from the included studies were analyzed using mean difference (MD) with 95% confidence intervals for consistent measurement tools, and standardized mean difference (SMD) with 95% confidence intervals for varying measurement tools. The analysis focused on effect measures related to pain and functional outcomes. If studies reported means and SDs for baseline and outcomes only, evidence-based medicine analysis methods were referenced (Ming, 2001; da Costa et al., 2013), the mean and SD of the change before and after were calculated, and when the number of intervention groups in the included literature was 3, the groups were combined based on the comparisons, as recommended by the Cochrane Handbook (Cumpston et al., 2019). Finally, the results were presented in two time periods (<2 months and ≧2 months).

## 3 Results

### 3.1 Study selection

**FIGURE 1:**
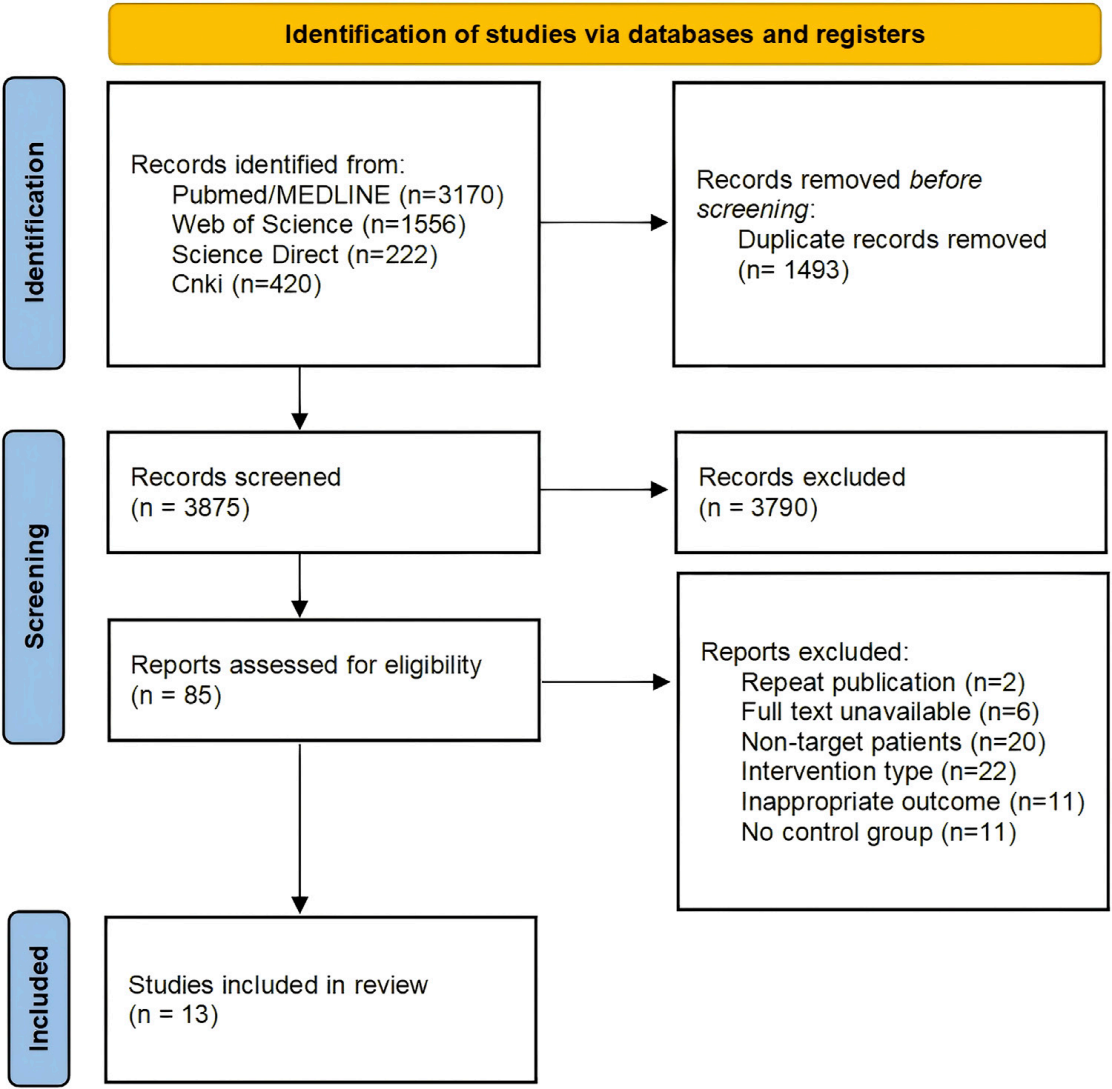
Flowchart of search and study selection.

The study screening flowchart is outlined by the PRISMA guidelines (Figure 1). Utilizing the specified keywords (including Mesh terms and synonyms), a comprehensive search yielded 5,368 records across multiple databases: Pubmed/MEDLINE (n = 3,170), Web of Science (n = 1,556), Science Direct (n = 222), and China Knowledge Network (CNKI) (n = 420). After duplicates were removed, 3,875 articles proceeded to initial screening. Following the review of titles and abstracts, 3,790 articles were excluded. A full-text assessment of the remaining 85 articles led to the inclusion of 13 studies, which featured 15 pairs of intervention and control groups, encompassing a total of 690 patients for the final analysis (Başkurt et al., 2011; Beaudreuil et al., 2011; Struyf et al., 2013; Hallgren et al., 2014; Dejaco et al., 2017; Boudreau et al., 2019; Gutiérrez-Espinoza et al., 2019; Tahran and Yeşilyaprak, 2020; Eliason et al., 2021; Macías-Hernández et al., 2021; AlAnazi et al., 2022; İğrek and Çolak, 2022; Gutiérrez Espinoza et al., 2023).

### 3.2 Characteristics of eligible studies

**FIGURE 2:**
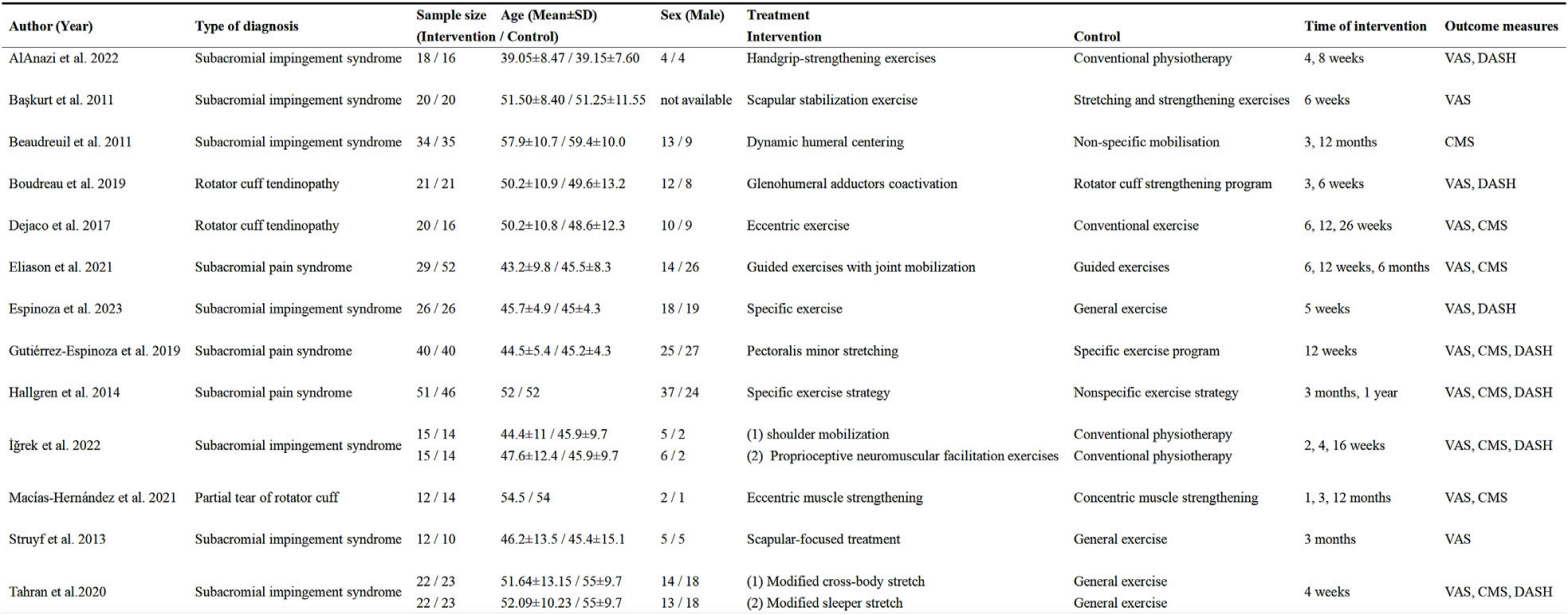
Characteristics of the studies included.

The basic characteristics and intervention details of the included literature are shown in Figure 2 and Supplementary Figure S1, including when the study was published, diagnostic type of the study population, sample size, age, gender, interventions and specific details, and time of assessment of outcomes and assessment metrics. Of the RCRSP patients included in the study, diagnosis types included subacromial impingement/pain (10 studies, n = 586) (Başkurt et al., 2011; Beaudreuil et al., 2011; Struyf et al., 2013; Hallgren et al., 2014; Gutiérrez-Espinoza et al., 2019; Tahran and Yeşilyaprak, 2020; Eliason et al., 2021; AlAnazi et al., 2022; İğrek and Çolak, 2022; Gutiérrez Espinoza et al., 2023), rotator cuff tendinopathy (2, n = 78) (Dejaco et al., 2017; Boudreau et al., 2019) and partial rotator cuff tear (1, n = 26) (Macías-Hernández et al., 2021), with a mean age of 49.1 years (range 39–59 years). In terms of interventions, one study used handgrip strength exercises (AlAnazi et al., 2022), five used scapular stabilization exercises (including dynamic humeral centering, glenohumeral adductor activation) (Başkurt et al., 2011; Beaudreuil et al., 2011; Struyf et al., 2013; Boudreau et al., 2019; Gutiérrez Espinoza et al., 2023), three used eccentric exercise (Hallgren et al., 2014; Dejaco et al., 2017; Macías-Hernández et al., 2021), five used joint mobilization/stretching (Gutiérrez-Espinoza et al., 2019; Tahran and Yeşilyaprak, 2020; Eliason et al., 2021; İğrek and Çolak, 2022) and one utilized proprioceptive neuromuscular facilitation exercises (İğrek and Çolak, 2022). Of the included studies, two were multicenter trials (Tahran and Yeşilyaprak, 2020; İğrek and Çolak, 2022), and pairwise comparisons were made between different intervention groups and the same control groups. Since there is no clear definition of the intervention period for rotator cuff-related shoulder pain, this article combines the previous literature and uses 2 months and 6 months as the intervention period for rotator cuff-related shoulder pain (Gebremariam et al., 2014; Hak et al., 2015; Lin et al., 2019). In this article, 2 months and 6 months were designated as the time points for short-term, mid-term, and long-term interventions. Five studies had follow-up periods both <2 months and ≧2 months (Dejaco et al., 2017; Eliason et al., 2021; Macías-Hernández et al., 2021; AlAnazi et al., 2022; İğrek and Çolak, 2022). Besides, four had long-term follow-up (>6 months) (Beaudreuil et al., 2011; Dejaco et al., 2017; Eliason et al., 2021; Macías-Hernández et al., 2021). However, the results of long-term follow-up were excluded from this analysis due to the substantial impact of participant attrition on the experimental outcomes. In addition to shoulder pain, functional scores were reported in 10 studies (Hallgren et al., 2014; Dejaco et al., 2017; Boudreau et al., 2019; Gutiérrez-Espinoza et al., 2019; Tahran and Yeşilyaprak, 2020; Eliason et al., 2021; Macías-Hernández et al., 2021; AlAnazi et al., 2022; İğrek and Çolak, 2022; Gutiérrez Espinoza et al., 2023), two studies only reported shoulder pain scores (Başkurt et al., 2011; Struyf et al., 2013), and one only functional scores (Beaudreuil et al., 2011).

### 3.3 Study quality and risk of bias

**FIGURE 3:**
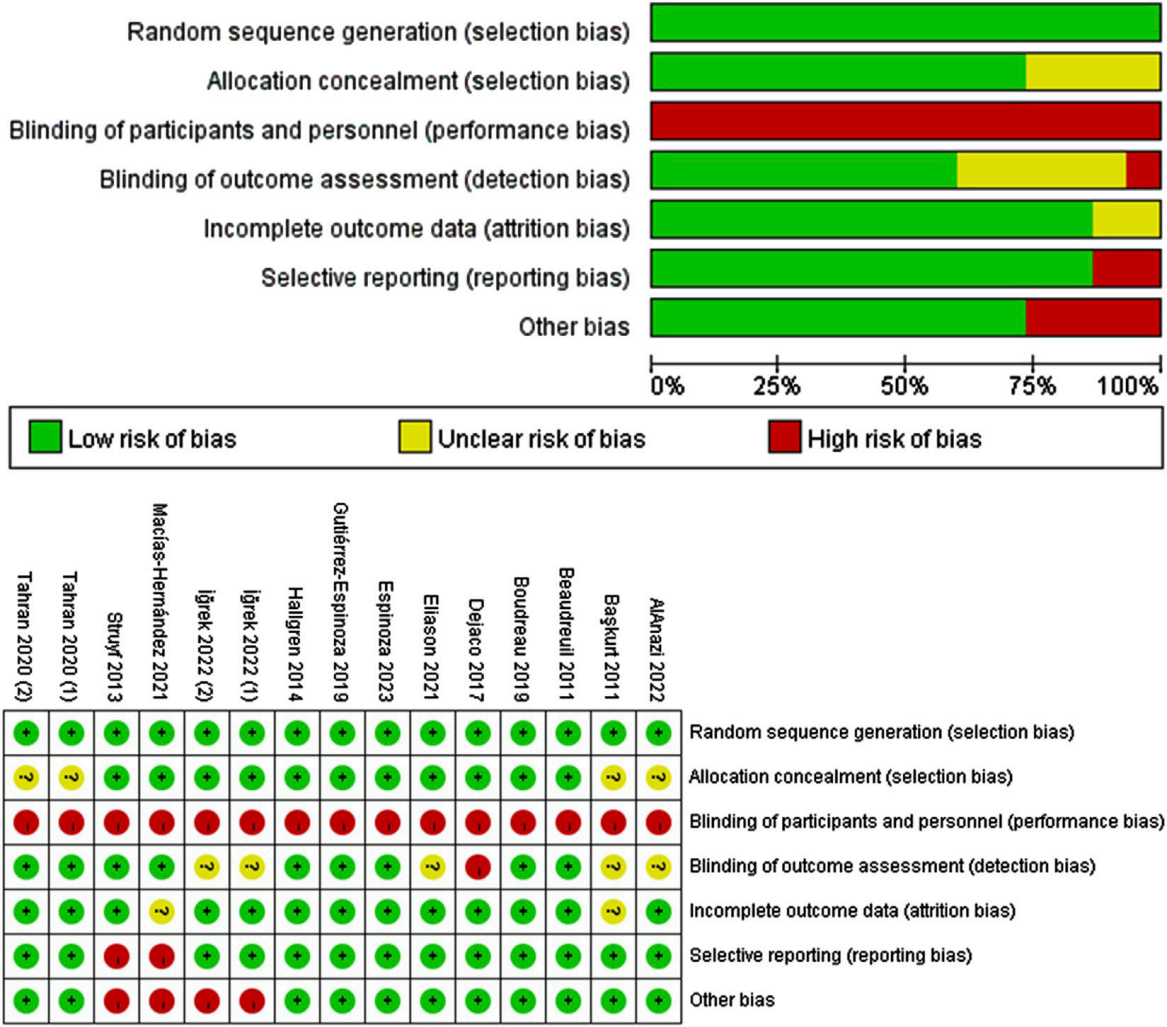
Risk of bias graph.

The distribution of risk of bias studies is generated according to the Cochrane Risk of Bias Tool and a summary table (Figure 3), with green representing low risk, yellow representing unclear risk, and red representing high risk. Overall, seven studies were of high quality (Beaudreuil et al., 2011; Hallgren et al., 2014; Boudreau et al., 2019; Gutiérrez-Espinoza et al., 2019; Tahran and Yeşilyaprak, 2020; Eliason et al., 2021; Gutiérrez Espinoza et al., 2023), four studies were of medium quality (Başkurt et al., 2011; Dejaco et al., 2017; AlAnazi et al., 2022; İğrek and Çolak, 2022) and two studies were of low quality (Struyf et al., 2013; Macías-Hernández et al., 2021). The most common methodological flaw was implementation bias, with all studies being high risk; followed by a small sample size (n < 30), with three being high risk (Struyf et al., 2013; Macías-Hernández et al., 2021; İğrek and Çolak, 2022), and then measurement bias, with four not reporting measurer blinding (Başkurt et al., 2011; Eliason et al., 2021; AlAnazi et al., 2022; İğrek and Çolak, 2022). Furthermore, one study was informed by the measurer (Dejaco et al., 2017). The overall methodological quality of the included literature was good in terms of selection bias (random allocation and allocation scheme concealment) and follow-up bias. The funnel plot scatter is roughly symmetrical (Supplementary Figure S2), indicating that publication bias was low in the included studies.

### 3.4 Synthesis of results

#### 3.4.1 Effects of specific exercise on pain

**FIGURE 4:**
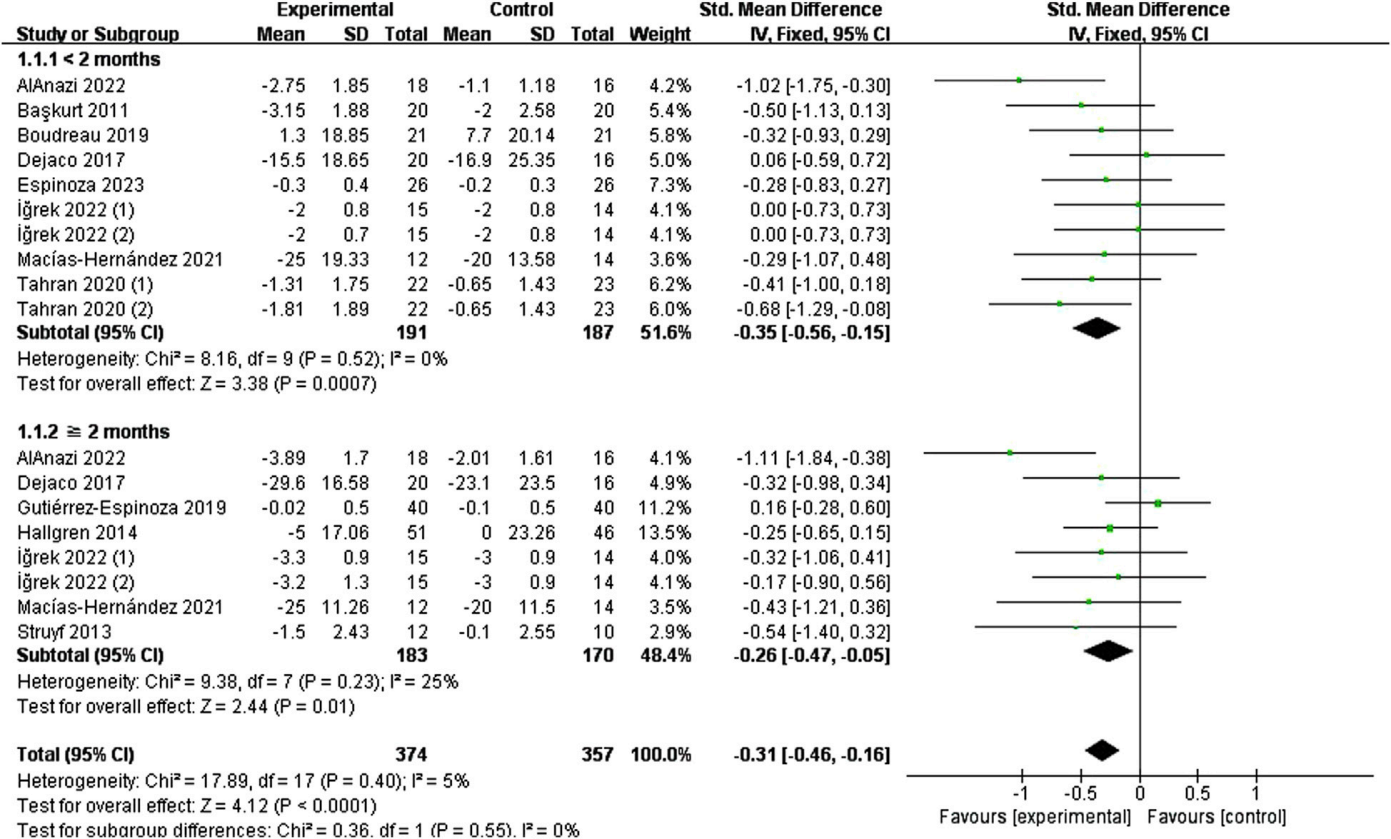
Effect of specific exercise intervention, compared with general exercise or routine physical therapy on shoulder pain over VAS.

Figure 4 is a forest plot of the pooled analysis of VAS pain scores (<2 months, 10 studies, n = 378; ≥2 months, eight studies, n = 353), which showed low heterogeneity across studies (I^2^ = 5%, P = 0.40), and combined effect sizes using a fixed-effects model, which indicated that exercise-specific strategies were more efficacious than non-specific exercises (SMD = −0.31, 95% CI: 0.46 to −0.16, P < 0.0001), but the effect was slight. Similarly, VAS scores were similarly lower in the intervention group than in the control group across the two time periods (<2 months (SMD = −0.35, 95% CI: 0.56 to −0.15, P = 0.0007); and ≥2 months (SMD = −0.26, 95% CI: 0.47 to −0.05, P = 0.01)), and the short-term effect was slightly superior to the mid-term effect.

#### 3.4.2 Effect of specific exercise on function

**FIGURE 5:**
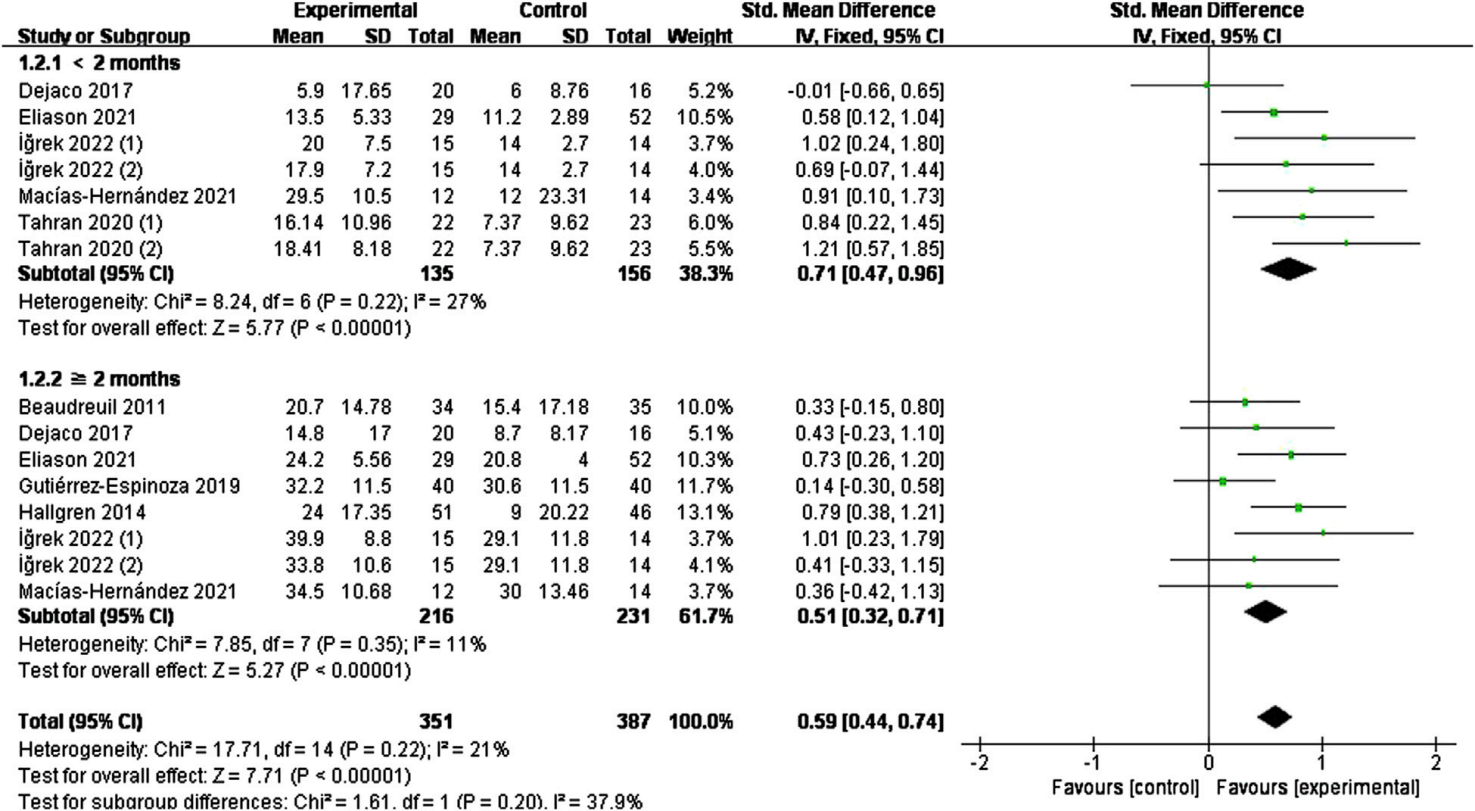
Effect of specific exercise intervention, compared with general exercise or routine physical therapy on shoulder function over CMS.

The effect of specific exercises on CMS function scores (<2 months, seven studies, n = 291; ≥2 months, eight studies, n = 447), with pooled analyses suggesting low heterogeneity across items (I^2^ = 21%, P = 0.22), and the combined effect sizes using a fixed-effects model, which showed that the intervention group was higher than the control group (SMD = 0.59, 95% CI: 0.44 to 0.74, P < 0.00001) (Figure 5). The results of the time subgroup analysis showed that the CMS scores of the intervention group were lower than those of the control group (<2 months (SMD = 0.71, 95% CI: 0.47 to 0.96, P < 0.00001); ≥2 months (SMD = 0.51, 95% CI: 0.32 to 0.71, P < 0.00001)), and that short-term effects were better than mid-term effects.

**FIGURE 6:**
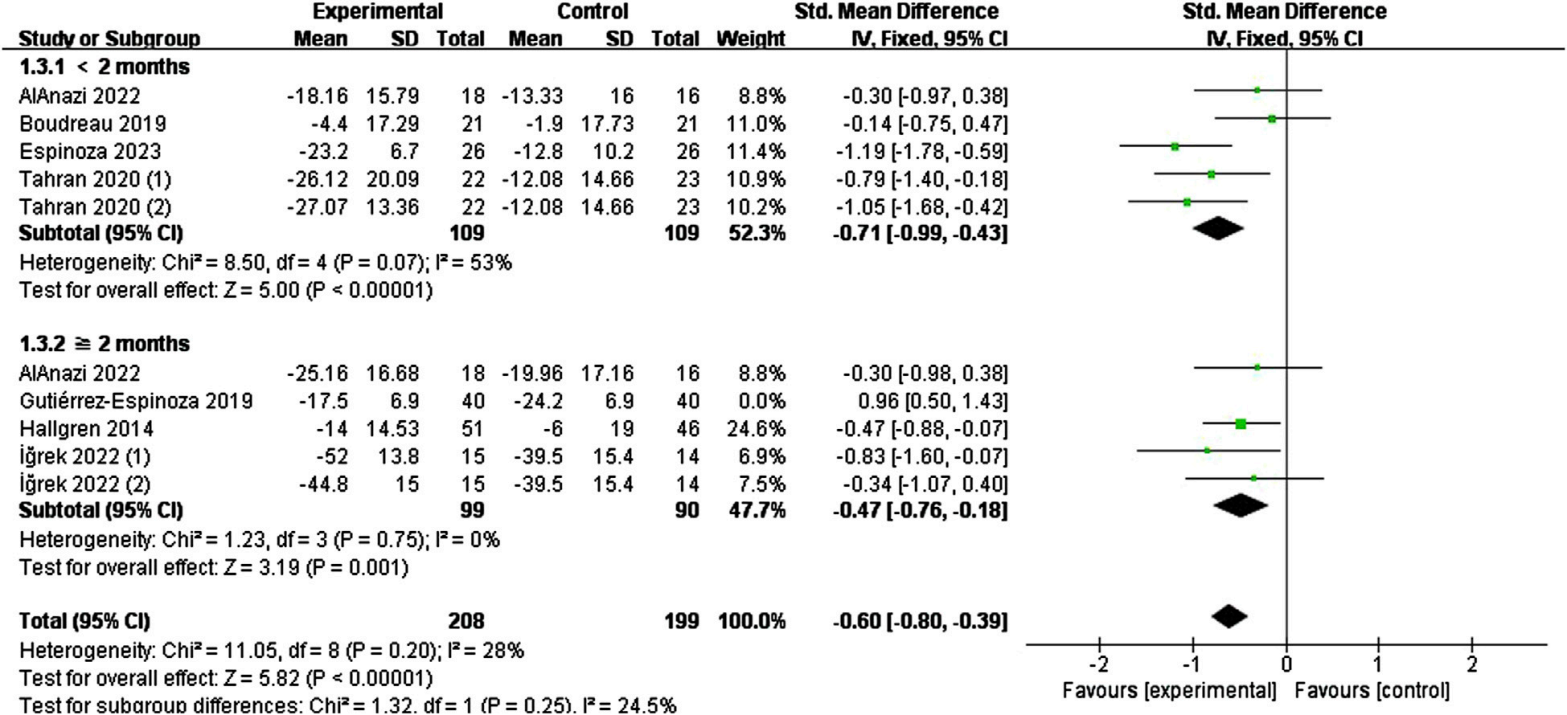
Effect of specific exercise intervention, compared with general exercise or routine physical therapy on shoulder function over DASH.

The effect of specific exercise on DASH function scores (<2 months, five studies, n = 218; ≥2 months, five studies, n = 269) takes high heterogeneity across studies (I^2^ = 81%, P < 0.00001). So we conducted a sensitivity analysis to identify the source of the heterogeneity by systematically removing each study. This analysis found it was originated from one study (Gutiérrez-Espinoza et al., 2019), which was subsequently excluded. Figure 6 shows the forest plot after exclusion, where heterogeneity was greatly reduced (I^2^ = 28%, P = 0.20). Combining effect sizes using a fixed model showed a reduction in DASH scores in the intervention group compared to the control group (SMD = −0.60, 95% CI: 0.80 to −0.39, P < 0.00001). Time subgroup analyses showed moderate heterogeneity in the <2 months subgroup (I^2^ = 53%, P = 0.07), no heterogeneity in the ≥2 months subgroup (I^2^ = 0%, P = 0.75). The intervention group demonstrated superior efficacy compared to the control in both time subgroups (short-term (SMD = −0.71, 95% CI: 0.99 to −0.43, P < 0.00001), mid-term (SMD = −0.47, 95% CI: 0.76 to −0.18, P = 0.001)). The short-term effect (<2 months) was similarly superior to the mid-term effect (≥2 months)

### 3.5 Subgroup analysis of the result

#### 3.5.1 Effects of different types of exercise on pain

**FIGURE 7:**
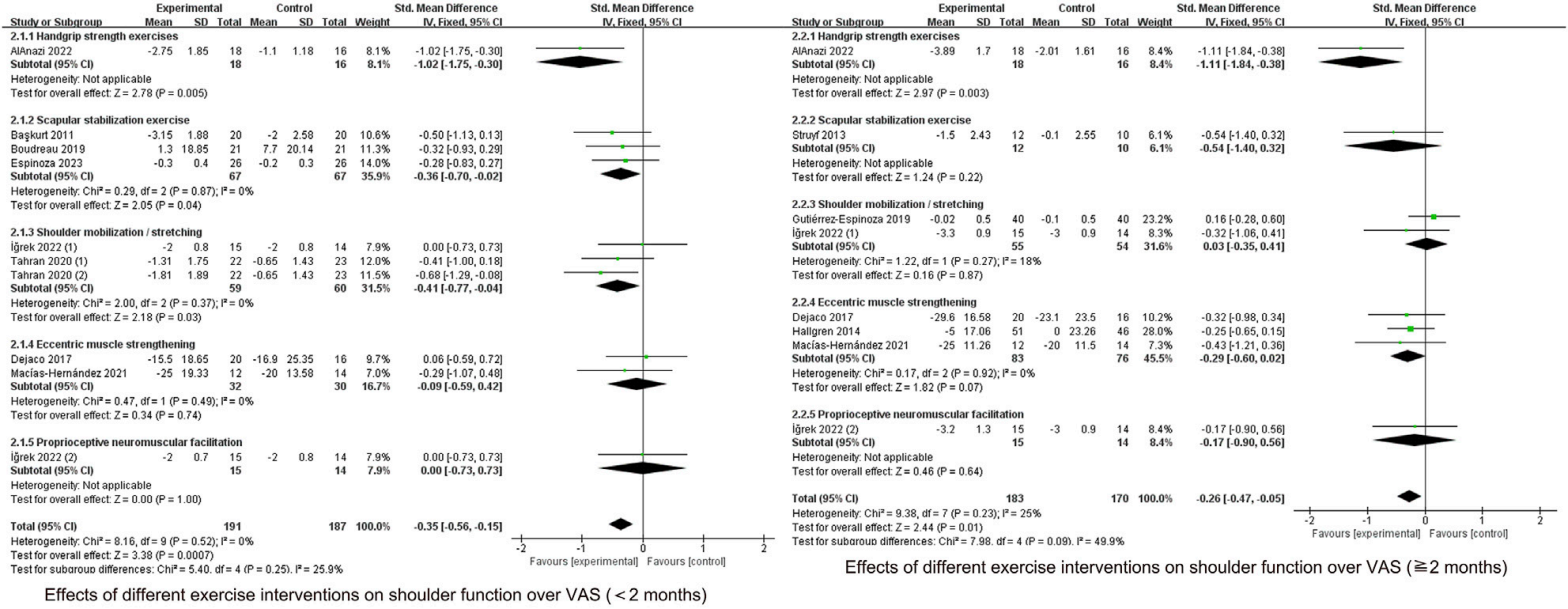
Effects of different exercise interventions on shoulder function over VAS.

As shown in Figures 3, 7 specific modes of exercise mildly improved short-term shoulder pain compared with general exercise/conventional physical therapy (handgrip strength exercises (SMD = −1.02, 95% CI: 1.75 to −0.30, P = 0.005), scapular stability training (SMD = −0.36, 95% CI: 0.70 to −0.02, P = 0.04), shoulder mobilization/stretching (SMD = −0.41, 95% CI: 0.77 to −0.04), P = 0.03)), whereas eccentric exercise (SMD = −0.09, 95% CI: 0.59 to −0.42), P = 0.74) and proprioceptive training (SMD = −0.00, 95% CI: 0.73 to 0.73, P = 1.00) did not show improvement.

At ≥2 months of follow-up, no significant improvement was seen in any of the four exercises except for handgrip strength exercises (SMD = −1.11, 95% CI: 1.84 to −0.38, P = 0.003).

#### 3.5.2 Effects of different types of exercise on function

**FIGURE 8:**
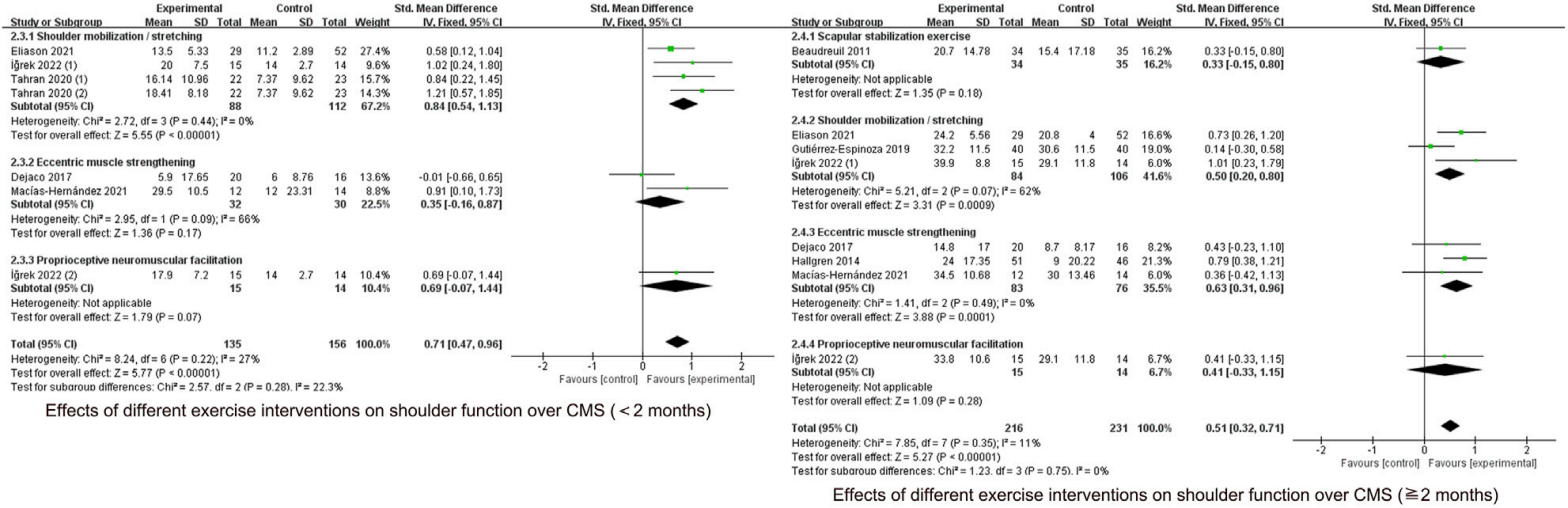
Effects of different exercise interventions on shoulder function over CMS.

CMS score subgroup analyses are shown in Figure 8. During the <2-month follow-up period, shoulder mobilization/stretching (SMD = 0.84, 95% CI: 0.54 to 1.13, P < 0.00001) improved CMS scores, whereas eccentric exercise (SMD = 0.35, 95% CI: 0.16 to 0.87, P = 0.17) and proprioceptive training (SMD = 0.69, 95%CI: 0.07 to 1.44, P = 0.07) did not show any improvement compared to the control group.

At ≥2-month follow-up, improvements were seen in shoulder mobilization/stretching (SMD = 0.50, 95% CI: 0.20 to 0.80, P = 0.0009) and eccentric exercise (SMD = 0.63, 95% CI: 0.31 to 0.96, P = 0.0001) whereas scapular stabilization exercises (SMD = 0.33, 95% CI: 0.15 to 0.80, P = 0.18) and proprioceptive training (SMD = 0.41, 95% CI: 0.33 to 1.15, P = 0.28) did not show improvement.

**FIGURE 9:**
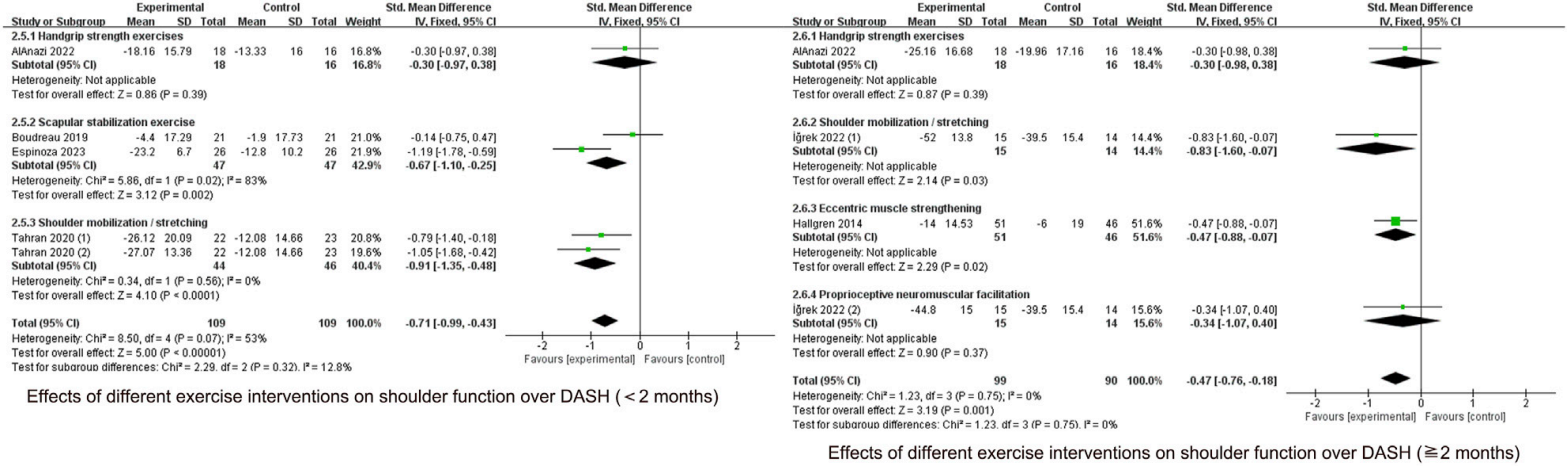
Effects of different exercise interventions on shoulder function over DASH.

As shown in Figure 9, scapular stabilization exercise (SMD = −0.67, 95% CI: 1.10 to −0.25, P = 0.002) and shoulder mobilization/stretching (SMD = −0.91, 95% CI: 1.35 to −0.48, P < 0.0001) improved function in the short term in the DASH score. Shoulder mobilization/stretching (SMD = −0.83, 95% CI: 1.60 to −0.07, P = 0.03) and eccentric exercise (SMD = −0.47, 95% CI: 0.88 to −0.07, P = 0.02) improved functional performance in the mid-term period, whereas other types of exercise did not.

## 4 Discussion

This review identified 13 randomized controlled trials (690 participants) comparing specific exercise therapy—defined as exercises targeting the activation and coordination of the scapular pectoralis muscles and/or the dynamic stabilizers of the humerus, including scapular stabilization, positioning, proprioception, neuromuscular control, strengthening, and stretching (Hanratty et al., 2012; Holmgren et al., 2014; Hanratty et al., 2016)— with conventional physical therapy or general exercise for rotator cuff-related shoulder pain. Specific exercise types and dosages were developed by clinicians and physical therapists. Our pooled analysis indicated that specific exercises improved shoulder pain (VAS) and function (CMS, DASH), though the effects were not pronounced and were slightly better within 2 months.

Subgroup analyses showed that handgrip strength exercises were effective in improving shoulder pain, but did not significantly improve function. Previous studies have revealed that the connection between handgrip strength and rotator cuff function is significant. A stronger handgrip boosts neural signals from the hand to the shoulder. Handgrip-strengthening exercises activate pathways that improve movement control and help integrate sensory information from both the shoulder and hand (Roberts et al., 2008; Kobesova et al., 2015). Besides, it decreases the activation of anterior portion of the deltoid, which contributes to impingement. No pain or functional improvement was seen with proprioceptive training compared to the control group, a finding that remains debatable due to the small study and sample size. On the other hand, proprioceptive training were proved effective in reducing pain in patients with partial supraspinatus tears in long-term follow-up, according to other research (Kim et al., 2015; Salles et al., 2015). This difference could be attributed to patient characteristics, variations in treatments, and differences in assessment methods between the studies. Scapular stabilization training and shoulder mobilization/stretching showed mild improvement in short-term shoulder pain, whereas an eccentric exercise program did not have a significant effect on short-term pain, and none of the three specific exercise types improved the pain level of the reduced shoulder after 2 months. Shoulder mobility/stretching had a positive effect on both CMS and DASH scores in the short to middle term, and eccentric exercise had an effect on the medium-term effect but not the short-term, and more evidence is still needed to demonstrate the effect of scapular stabilization exercises on shoulder function in the short to medium term. Research has shown that the position of the humeral head and scapula affects the width of the subacromial space. As a result, strengthening the depressor muscles, such as the subscapularis, infraspinatus, and teres minor, is crucial (Faber et al., 2006; Camargo et al., 2009). Therefore, shoulder rehabilitation should incorporate scapular stabilization training as well as shoulder mobilization and stretching exercises. One review supports our view, indicating that eccentric exercise offers a slightly better reduction in pain compared to other exercises, though it does not significantly improve function, especially during middle to long-term follow-up (Larsson et al., 2019). On the other hand, incorporating eccentric loads, which are not limited by concentric strength, seem to be more effective than traditional resistance training for enhancing strength, power, and speed performance (Douglas et al., 2017).

The primary factor affecting the quality of the included literature is implementation bias, as all studies are considered high-risk. Due to the specificity of exercise interventions, the exercise programs for different subjects must be continually evaluated and adjusted, making it unrealistic to blind researchers to group assignments. In eight studies, the evaluators were blinded, while one was not, which may lead to distortions in the study results (Chen et al., 2022). Additionally, the second issue is the insufficient sample size, which fails to accurately reflect the overall situation. In the data collection and organization, baseline and outcome score data were given in each of the eight articles (Başkurt et al., 2011; Beaudreuil et al., 2011; Struyf et al., 2013; Hallgren et al., 2014; Dejaco et al., 2017; Eliason et al., 2021; Macías-Hernández et al., 2021; AlAnazi et al., 2022). In order to further assess the effect of the intervention, statistical methods were used to calculate the mean and SD of the before and after changes which may also have some impact on the outcome (da Costa et al., 2013).

Forest plots evaluating DASH function revealed substantial heterogeneity among studies, particularly at the 2-month time point. This heterogeneity was significantly reduced following sensitivity analysis through item-by-item exclusion and subsequent subgroup analyses (Figure 6).

Previous reviews and guidelines have recommended exercise as a therapeutic approach through a variety of mechanisms, including neuromuscular synergism that can reduce pain and improve function in patients with RCRSP (Diercks et al., 2014; Holmgren et al., 2014; Powell et al., 2022). Based on the latest scientific literature and clinical experience, exercise is best performed at low intensity and high frequency, within the pain threshold, with a focus on eccentric exercise to strengthen the rotator cuff and eccentric exercise to strengthen the scapular stabilizers, with specific exercise strategies that can be defined as the activation and coordination of the shoulder-thoracic musculature or movements involving the dynamic humeral stabilizers of the shoulder joint. These exercises include scapular stabilization, postural adjustments, proprioception, neuromuscular control, strength training, and stretching, and the exercises must involve some form of resistance such as one’s own body weight, elastic resistance, weight-bearing equipment, dumbbells, and/or strength training machines.

Aside from the specific exercise modalities, the role of the physiotherapist is often overlooked. However, the physiotherapist can unintentionally influence patients' perceptions of their treatment and even their decisions regarding surgery. As therapists vary in their treatment modalities and patients' physical conditions and rehabilitation goals, the above exercise methods need to be personalized through individualization (Bennell et al., 2014) This reflects the importance of applying exercise prescription (Crookham, 2013; Sijia et al., 2023), which serves as a vehicle for exercise therapy and can specify parameters such as the patient’s exercise modality and dosage, greatly increasing the efficacy of exercise interventions. Although there is a large amount of literature related to the application of exercise therapy in RCRSP, there is a lack of documentation on the prescription of specific exercise modalities and dosages, which has resulted in the existence of limitations in the development of exercise prescription in China, which is currently more widely used in the study of common chronic diseases in middle-aged and old-aged people (e.g., hypertension, diabetes mellitus, osteoarthritis, etc.) (Pedersen and Saltin, 2015; Yuge and Zhenguo, 2021) and fewer studies in shoulder injury diseases. Inspiringly, the publication of an expert consensus to further clarify the definition, development, and implementation process of exercise prescription may help resolve this difficulty (Yuefeng, 2023).

The specific parameters of the exercise are also crucial, and they must be aligned with standardized prescription reports that include detailed intervention information. A study has shown that the Consensus on Exercise Intervention Reporting Template (CERT) was developed specifically to address the issue of incomplete reporting of exercise interventions (Kucksdorf et al., 2024). However, the completeness of reporting of exercise intervention content is currently poor in research trials in patients with rotator cuff disease. CERT is a reliable tool for assessing the completeness of reporting of exercise interventions in trials. Future research should be based on CERT for the assessment of outcomes and reporting of exercise protocols, which can greatly contribute to the analyzability and generalizability of research results.

The results of this article suggest that specific modes of exercise are effective in improving pain levels and functional status in rotator cuff-related shoulder pain, but they do not demonstrate a significant advantage over general exercise or conventional physical therapy. The results of other reviews have shown that eccentric exercise and scapular stability training can lead to a slight reduction in pain levels in patients with subacromial impingement syndrome, but with little clinical significance (Larsson et al., 2019; Naunton et al., 2020; Ravichandran et al., 2020), which is consistent with the findings of this article. Combining the results from the subgroup analysis of intervention types suggests that incorporating shoulder-specific exercise therapies into a tailored shoulder rehabilitation program could be effective. Implementing these therapies through targeted exercise prescriptions, addressing multiple areas, may enhance pain relief and functional improvement for optimal clinical outcomes.

This review has several notable strengths. Firstly, it encompasses a broad range of exercises, incorporating a diverse array of specific intervention types. Secondly, unlike prior studies that have focused solely on subacromial impingement, rotator cuff tendinopathy, or rotator cuff injuries in isolation, this review is the first to examine the overall impact on the efficacy of RCRSP. In clinical practice, pain is usually the main reason for RCRSP patients to visit the clinic (van der Windt et al., 1996; Mitchell et al., 2005), and our results provide a new reference for the development and implementation of appropriate exercise prescriptions. Individualized, supervised, progressive, and targeted exercise regimens based on prescriptions could be a new option in addition to surgery, corticosteroid injections, and physical therapy (Dubé et al., 2023).

This article identifies the efficacy of specific exercises, but there are still some limitations. Due to individual differences, such as varying rehabilitation goals and physical conditions among patients, it is unrealistic to establish uniform standards for exercise types and dosages. These factors contribute to the challenges of applying specific exercises in a clinical setting. Among the articles included in this study, two articles were vague in their descriptions of intervention details (Dejaco et al., 2017; Eliason et al., 2021), and the rest of the articles did not establish corresponding exercise dosages norms, such as insufficient description of key features like pain during loading, duration of exercise, rest time between sets of exercise, and the rhythm of exercise. As exercise dosage is another important component that has a status comparable to that of exercise type (Bennell et al., 2014; Holmgren et al., 2014), a full understanding can help to obtain convincing study results. Secondly, participant attrition results in the failure to analyze long-term efficacy (>6 months). Potential reasons include patient crossover to surgical interventions and high participant mobility, particularly in multicenter trials. Only five of the 13 studies included in the analysis reported data beyond 6 months, with small sample sizes. Despite the limited evidence, we advocate for phased exercise prescriptions in clinical practice, with a focus on maintenance programs to ensure sustained benefits. For outcome measurement, rationale for subjective metrics (VAS, CMS, and DASH) are validated tools with well-established minimal clinically important differences (MCID) suitable for meta-analysis pooling. These metrics directly reflect patients’ perceptions of functional recovery and quality of life. Though we recognize the benefits of adding objective measurements, these outcome indicators are diverse. The movement of joints occurs in multiple directions, different studies may describe ROM in diverse ways due to these variations. On the other hand, strength testing protocols vary significantly, from isokinetic testing to manual resistance. Imaging findings, such as MRI measurements of tendon thickness, show a poor correlation with clinical symptoms, and there are no standardized imaging scores available yet.

## 5 Conclusion

Exercise is frequently recommended as a treatment for chronic sports injuries according to previous review guidelines. This article demonstrates that specific modes of exercise for the shoulder can enhance both pain relief and functional status in patients with Rotator Cuff-Related Shoulder Pain (RCRSP). While these targeted exercises offer valuable insights for clinical shoulder rehabilitation prescriptions, they do not show a obvious therapeutic advantage over general exercise or conventional physical therapy. Furthermore, the lack of standardized and effective exercise reporting across studies constrains the practical application and development of exercise prescriptions in clinical setting.

## Data Availability

The original contributions presented in the study are included in the article/Supplementary Material, further inquiries can be directed to the corresponding author.
