# Supplementary material for: Specific modes of exercise to improve rotator cuff-related shoulder pain: systematic review and meta-analysis
Source: Front Bioeng Biotechnol. 2025 Apr 8;13:1560597. doi: 10.3389/fbioe.2025.1560597 (PMC12011739; doi:10.3389/fbioe.2025.1560597)
Supplement: Supplementary file 1 [file Table1.docx]

**Search strategy**

**Pubmed:**

((("exercise therapy"[MeSH Terms] OR "exercise therapy"[All Fields]) OR ("exercise"[MeSH Terms] OR "exercise"[All Fields]) OR ("exercise prescription"[MeSH Terms] OR "exercise prescription"[All Fields]) OR ("training"[MeSH Terms] OR "training"[All Fields]) OR ("kinematic"[MeSH Terms] OR "kinematic"[All Fields])) AND (("rotator cuff related shoulder pain"[MeSH Terms] OR "rotator cuff related shoulder pain"[All Fields]) OR ("subacromial impingement syndrome"[MeSH Terms] OR "subacromial impingement syndrome"[All Fields]) OR ("rotator cuff injury"[MeSH Terms] OR "rotator cuff injury"[All Fields]) OR ("rotator cuff tendinopathy"[MeSH Terms] OR "rotator cuff tendinopathy"[All Fields])))

**Web of Science:**

TS=(exercise therapy OR exercise OR exercise prescription OR training OR kinematic) AND TS=(rotator cuff related shoulder pain OR subacromial impingement syndrome OR rotator cuff injury OR rotator cuff tendinopathy)

**Science Direct:**

(exercise therapy OR exercise OR exercise prescription OR training OR kinematic) AND (rotator cuff related shoulder pain OR subacromial impingement syndrome OR rotator cuff injury OR rotator cuff tendinopathy)

**Cochrane:**

#1 (exercise therapy OR exercise OR exercise prescription OR training OR kinematic)

#2 (rotator cuff related shoulder pain OR subacromial impingement syndrome OR rotator cuff injury OR rotator cuff tendinopathy)

#3 #1 AND #2

**Cnki:**

(SU%='运动疗法' OR SU%='锻炼' OR SU%='运动处方' OR SU%='训练' OR SU%='运动') AND (SU%='肩袖相关肩痛' OR SU%='肩峰下疼痛' OR SU%='肩袖损伤' OR SU%='肩袖肌腱病')
