# Supplementary figures and images for: Specific modes of exercise to improve rotator cuff-related shoulder pain: systematic review and meta-analysis
Source: Front Bioeng Biotechnol. 2025 Apr 8;13:1560597. doi: 10.3389/fbioe.2025.1560597 (PMC12011739; doi:10.3389/fbioe.2025.1560597)

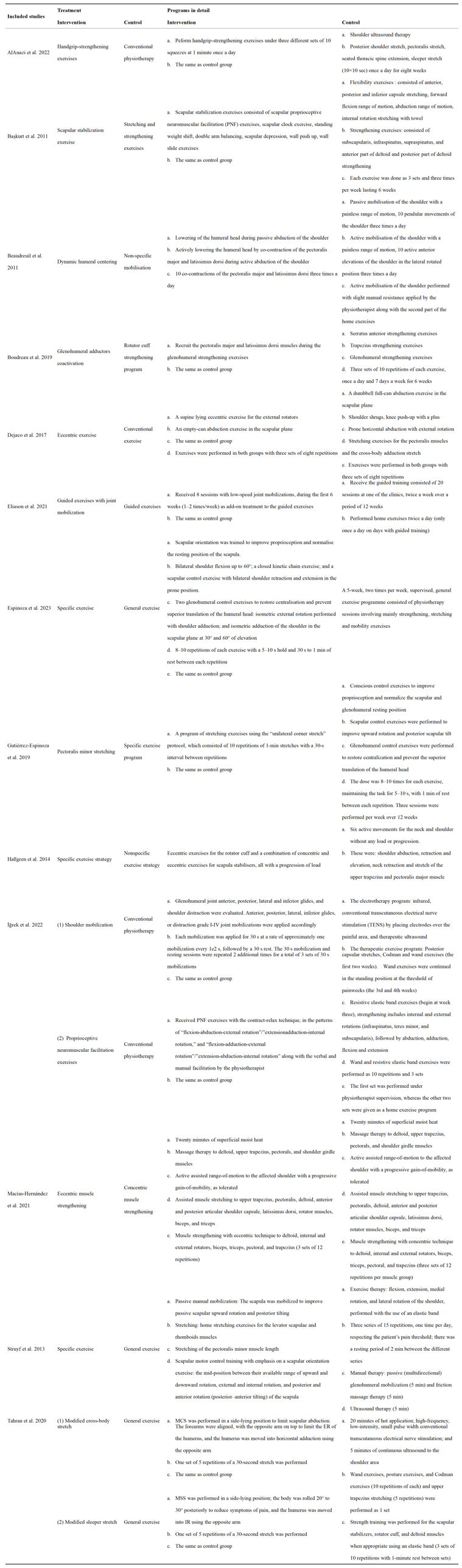

Supplement: Supplementary file 2 [file Image1.jpeg]

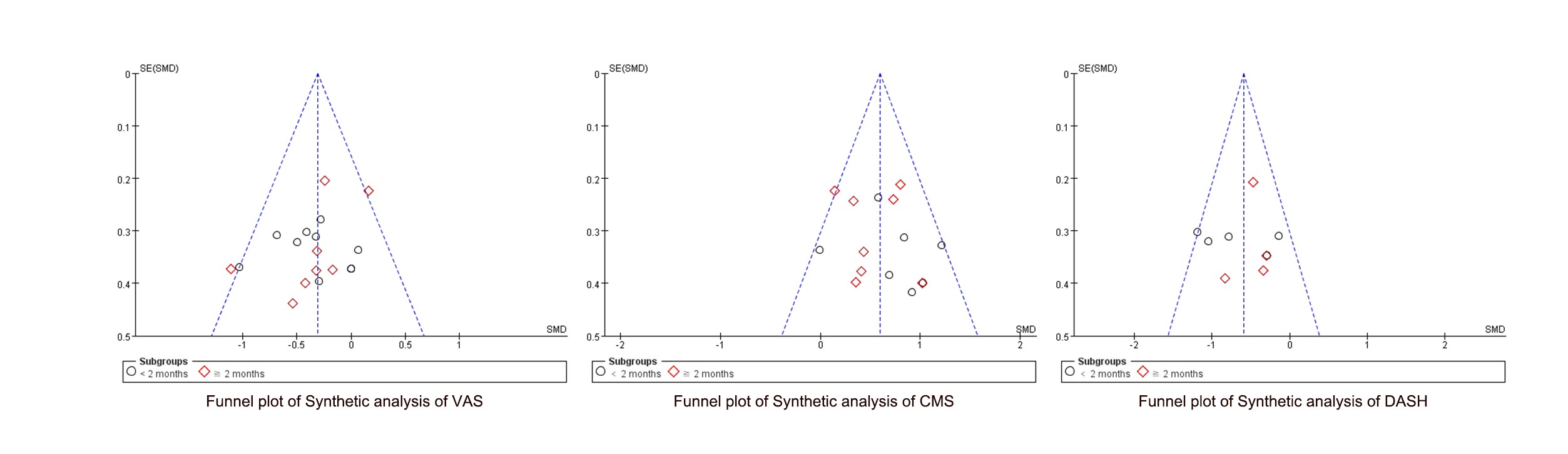

Supplement: Supplementary file 3 [file Image2.jpeg]
